# Pulmonary IgG4‐related disease with favourable response to rituximab: A case report

**DOI:** 10.1002/rcr2.1061

**Published:** 2022-11-01

**Authors:** Romain Muller, Mikael Ebbo, Paul Habert, Julia Torrents, Jean Yves Gaubert, Nicolas Schleinitz

**Affiliations:** ^1^ Internal Medicine Department Hopital La Timone, APHM, Aix Marseille University Marseille France; ^2^ Imaging Department Hopital Nord, APHM, Aix Marseille University Marseille France; ^3^ Anatomopathology Department APHM, Aix Marseille University Marseille France; ^4^ Imaging Department Hopital La Timone, APHM, Aix Marseille University Marseille France

**Keywords:** interstitial lung disease, rare lung diseases

## Abstract

Pulmonary involvement of IgG4‐associated disease is a rare condition with no codified treatment apart from steroid administration. We report here the case of a patient with pulmonary involvement of IgG4‐RD successfully managed with Rituximab, in induction and maintenance therapy. This original case could support the use of Rituximab in rare situations of steroid‐resistant or steroid‐dependent pulmonary IgG4‐RD.

## Graphical abstract

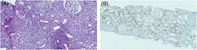

## INTRODUCTION

IgG4‐related disease (IgG4‐RD) is a systemic orphan disease that can affect almost all organs. It is characterized by unifying histopathological fibroinflammatory lesions.^1^ The most frequent organ involved are pancreas, bile ducts, salivary and lachrymal glands, retroperitoneum, lungs, kidneys, and aorta. Pulmonary involvement is relatively less frequent but is probably underestimated. Observational studies have shown that radiological presentation of pulmonary involvement in IgG4‐RD is polymorphic and often non‐specific.^2^

The disease is usually treated by steroids, with a remarkable efficacy but relapses are frequent. Second line treatments as monoclonal anti‐CD20 antibodies are usually proposed in patients with relapses as steroid sparing agents. We report here a case of IgG4‐RD with pulmonary involvement and his response to treatment.

## CASE REPORT

**FIGURE 1:**
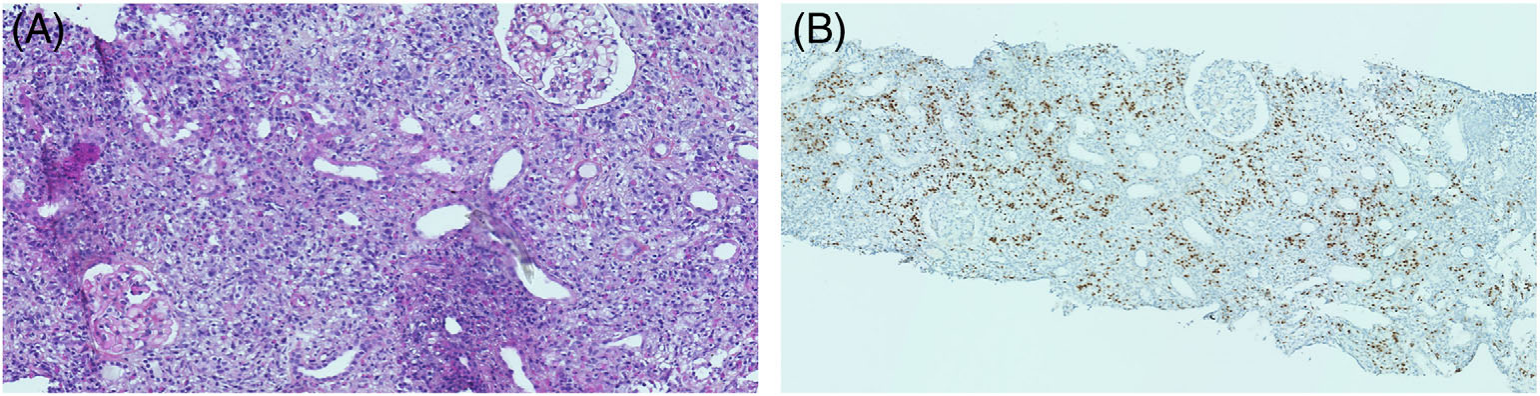
Renal biopsy of the patient. (A) Renal lymphocytic infiltrate with few eosinophils and fibrotic lesions (HPS stain). (B) IgG4 labelling (immunohistochemistry): abundant IgG4 plasma cells

**FIGURE 2:**
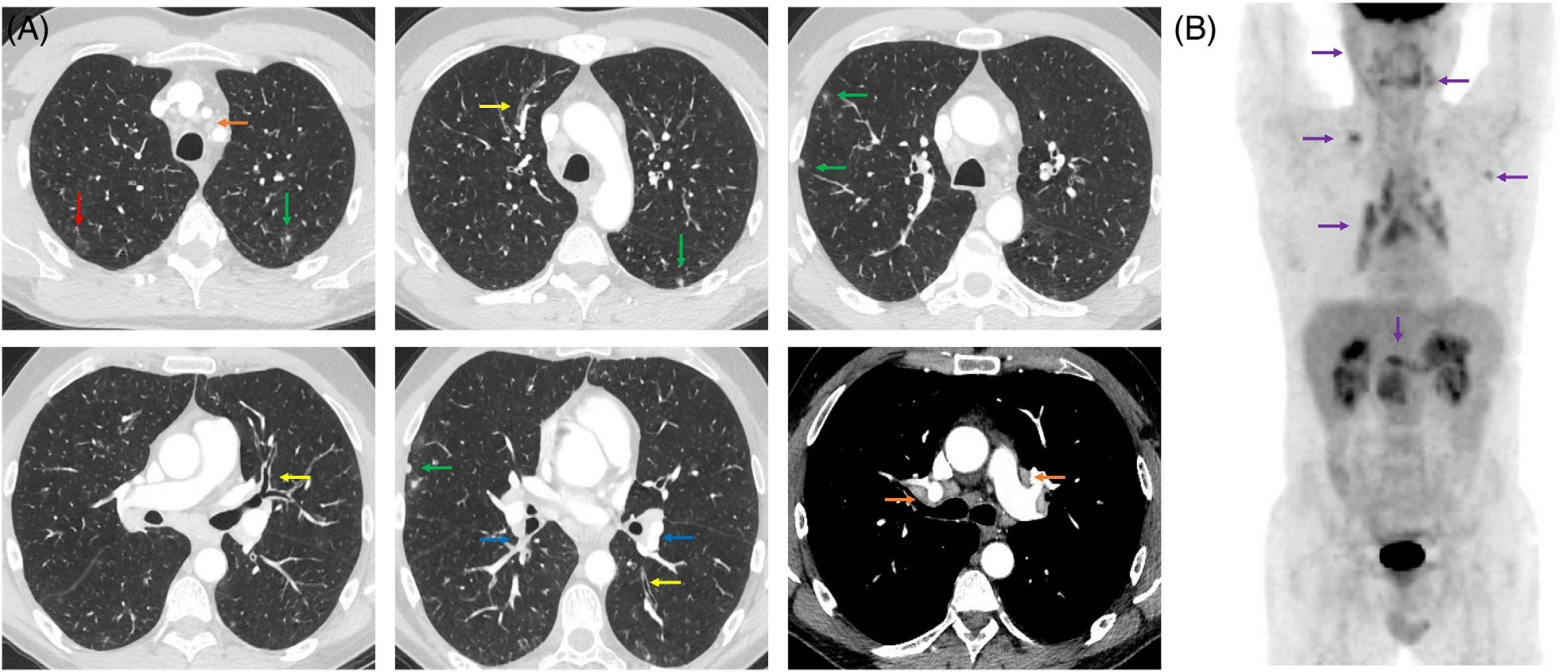
(A) Chest CT‐scan at diagnosis: multiple and heterogeneous pulmonary lesions of IgG4‐RD: Red arrow: ground glass area; Orange arrow: lymphadenopathies; Green arrows: nodules mainly sub‐pleural; Yellow arrows: bronchial wall thickening; Blue arrows: peribronchovascular thickening. (B) Body PET‐scan before Rituximab. Purple arrows: Increased ^18^FDG uptake (PET scan) of salivary glands, mediastinum, pulmonary nodules and pancreas

**FIGURE 3:**
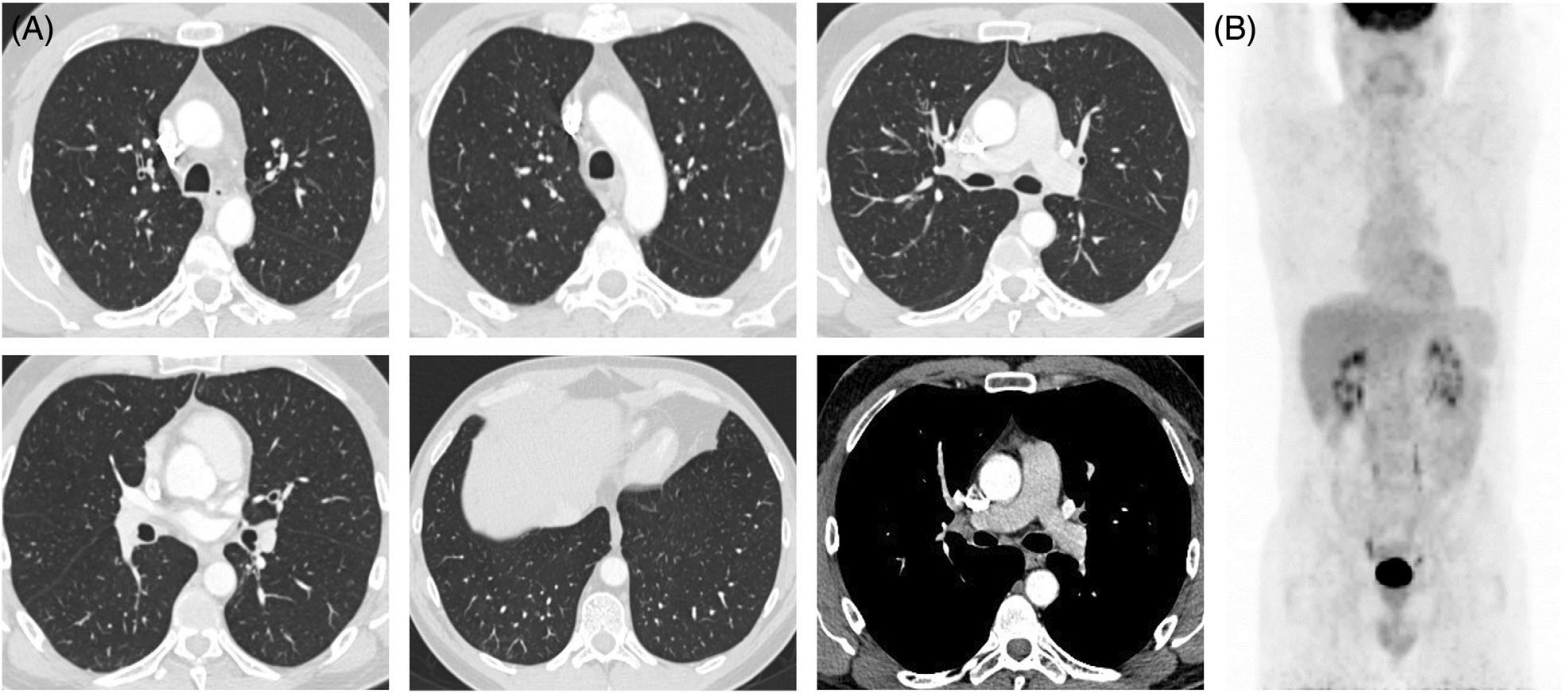
Chest CT‐scan (A) and body PET‐scan (B) after rituximab: Full regression of lung lesions

A 35‐year‐old male patient with no previous medical history presented with asthenia associated with submaxillitis and abdominal pain persisting for 2 months. Biological workup identified an inflammatory syndrome (C reactive protein 43 mg/dL, N < 5), moderate renal failure (creatinine 148 μg/ml), increased lipase (302UI/L, N < 60) and IgG4 serum levels (4.7 g/L, N < 1). Flow cytometry showed increased circulating plasmablast counts (11,745/ml). Body CT scan revealed numerous lymphadenopathies, diffuse enlargement of the pancreas and homogenous hypertrophy of the submaxillary glands. Renal biopsy showed a dense lymphocytic infiltrate with a ratio of IgG4+ plasma cells to total plasma cells of 50% and 40 IgG4+ plasma cells per HPF confirming the diagnosis of IgG4‐RD (Figure 1). Chest CT scan identified numerous mediastinal adenopathies, associated with peribronchovascular thickening, multiple pulmonary nodules and ground glass areas (Figure 2A). The patient was treated with 0.6 mg/kg prednisone in combination with azathioprine. He achieved complete clinical, biological and radiological remission at 3 months, including normalization of serum IgG4 and plasmablast levels. Steroids were gradually reduced and stopped after 1 year of follow‐up and azathioprine was continued as the sole maintenance treatment. Six months after steroids discontinuation, while still receiving azathioprine, the patient complained of asthenia, cough and sub‐maxillary glandular hypertrophy. CT‐scan revealed reappraisal of pulmonary and submaxilar lesions, and whole‐body TEP‐scanner revealed increased ^18^FDG uptake of salivary glands, mediastinum, pulmonary nodules and pancreas (Figure 2B). Biological evaluation showed increased serum CRP (29 mg/dL) and IgG4 (3,2 g/L) levels, associated with high levels of circulating plasmablasts (8029/mL). Steroid therapy was reintroduced at 30 mg/day, resulting in only partial improvement after 1 month. Rituximab (1 g twice day 1 and 15) was initiated, leading to complete remission at 3 months, including lung manifestations (Figure 3). For remission maintenance he received Rituximab 500 mg every 6 month for 2 years allowing steroid withdrawal. After 2 years of follow‐up the patient remained in complete, biological, clinical and radiological remission.

## DISCUSSION

We report here the original case of a patient with multiorgan IgG4‐RD involvement, including lung.

The diagnosis of IgG4‐RD requires ideally pathological confirmation because elevation of serum IgG4 is not specific and can be absent. The diagnosis is retained after a workup with the aim to exclude disease mimickers. Classification criteria for IgG4‐RD have been reported recently. Although there are not necessary for the diagnosis they listed exclusion criteria's and when these are absent a scoring by organ that include thoracic radiological abnormalities.^1^ A score over 20 is associated with a specificity and a sensitivity of 99 and 85% respectively. The case reported here presented with an ACR/EULAR score of 33. In this case lung involvement was polymorphic and associated various described pattern of the disease, as reported in IgG4‐RD. Pulmonary lesions were not investigated by biopsy, because they occurred in the context of multiorgan involvement with typical pathological lesions on kidney biopsy. Based on the response to treatment, lung lesions may be retrospectively classified as ‘highly probable’ IgG4‐RD‐related pulmonary involvement according to Corcoran et al.^3^

There are very sparse reports on efficacy of Rituximab in IgG4‐RD pulmonary involvement. Two main studies have reported the efficacy of anti‐CD20 in IgG4‐RD: Carruthers et al.^4^ and Ebbo et al.^5^ The overall response rate of patients was 97 and 93.5%, respectively. However, these overall response rates do not provide specific information on the evolution of the pulmonary lesions eventually presented by the patients. Large descriptive series of lung lesions are most often focused on the initial presentation of the disease, without describing their evolution under treatment.

In conclusion, we report here a case of a patient with ‘highly probable’ lung involvement according to Corcoran et al., illustrating the heterogeneous radiological pattern that can be observed in IgG4‐RD and the response to treatment by steroid and rituximab.

## AUTHOR CONTRIBUTIONS

Romain Muller, Mikael Ebbo and Nicolas Schleinitz concepted and draft the manuscript. Paul Habert, Julia Torrents and Jean Yves Gaubert revised it.

## CONFLICT OF INTEREST

None declared.

## ETHICS STATEMENT

The authors declare that appropriate written informed consent was obtained for the publication of this manuscript and accompanying images.

## Data Availability

The data that support the findings of this case report are available from the corresponding author upon reasonable request.
